# Pharmacokinetics of Nafamostat, a Potent Serine Protease Inhibitor, by a Novel LC-MS/MS Analysis

**DOI:** 10.3390/molecules27061881

**Published:** 2022-03-14

**Authors:** Hyeon Seok Oh, Taehyung Kim, Dong-Hyeon Gu, Tae Suk Lee, Tae Hwan Kim, Soyoung Shin, Beom Soo Shin

**Affiliations:** 1 School of Pharmacy, Sungkyunkwan University, Suwon 16419, Korea; berta0821@skku.edu (H.S.O.); 0108kth@skku.edu (T.K.); kdh951029@skku.edu (D.-H.G.); ts7619@skku.edu (T.S.L.); 2 College of Pharmacy, Daegu Catholic University, Gyeongsan 38430, Korea; thkim@cu.ac.kr; 3 College of Pharmacy, Wonkwang University, Iksan 54538, Korea; shins@wku.ac.kr

**Keywords:** nafamostat, COVID-19, LC-MS/MS, pharmacokinetics, bioavailability

## Abstract

Nafamostat, a synthetic serine protease inhibitor, has been used for the treatment of inflammatory diseases such as pancreatitis. Recently, an increasing number of studies have shown the promising antiviral effects of nafamostat for the treatment of coronavirus disease-19 (COVID-19). This study aimed to develop a novel liquid chromatography–tandem mass spectrometry (LC-MS/MS) analysis and to characterize the pharmacokinetics of nafamostat in rats. Nafamostat in the rat plasma was extracted by solid phase extraction, and ^13^C_6_-nafamostat was used as an internal standard. The quantification limit of nafamostat in the rat plasma was 0.5 ng/mL. The LC-MS/MS method was fully validated and applied to characterize the pharmacokinetics of nafamostat in rats. Following intravenous injection (2 mg/kg), nafamostat in the plasma showed a multiexponential decline with an average elimination half-life (t_1/2_) of 1.39 h. Following oral administration of nafamostat solutions (20 mg/kg) in 10% dimethyl sulfoxide (DMSO) and in 10% DMSO with 10% Tween 80, nafamostat was rapidly absorbed, and the average oral bioavailability was 0.95% and 1.59%, respectively. The LC-MS/MS method and the pharmacokinetic information of nafamostat could be helpful for the further preclinical and clinical studies of nafamostat.

## 1. Introduction

Nafamostat, a potent serine protease inhibitor, acts as a fast-acting proteolytic inhibitor, which inhibits a wide spectrum of proteases. By inhibiting thrombin and plasmin, nafamostat prevents the proteolysis of fibrinogen into fibrin [1], and is used as an anticoagulant during continuous venous hemodialysis to reduce hemorrhagic complications [2,3]. Inhibiting trypsin and kallikrein, nafamostat is also used for the treatment of acute pancreatitis [4,5]. Moreover, nafamostat has shown antitumor effects either alone or in combination with other therapies against various malignant tumors such as pancreatic and colorectal cancers [6].

Recently, an increasing number of studies have shown the potent antiviral activities of nafamostat on coronavirus disease-19 (COVID-19) caused by severe acute respiratory syndrome coronavirus 2 (SARS-CoV-2) [7,8,9,10,11]. There are also several clinical trials ongoing to evaluate the efficacy of nafamostat in patients with COVID-19 (ClinicalTrials.gov identifier: NCT04418128, NCT04352400, NCT04390594). Viral invasion by SARS-CoV-2, a single-stranded RNA-enveloped virus, is mediated by the structural spike (S) protein on its surface [12,13]. For entry into the host cells, SARS-CoV-2 binds to membrane-bound angiotensin-converting enzyme 2 (ACE2) receptor [12,13]. During infection, the S protein is cleaved into subunits, S1 and S2, by cellular proteases, and S1 allows SARS-CoV-2 to directly bind to the peptidase domain of the ACE2 receptor. S2 is further cleaved by a host type 2 transmembrane serine protease 2 (TMPRSS2), resulting in the fusion between the viral envelope and cell membrane and viral entry into human cells. Nafamostat is a TMPRSS2 inhibitor. Nafamostat has been shown to inhibit SARS-CoV-2 entry into host cells and block SARS-CoV-2 infection of human lung cells [7]. Nafamostat is expected to bind spontaneously and stably to the catalytic center of TMPRSS2 and inhibit its proteolytic processing of the S protein [7,10,11,14,15]. However, the effect of nafamostat on COVID-19 is still controversial. In a recently completed Phase 2 clinical trial (NCT04623021), no significant clinical improvement was observed in hospitalized patients with moderate to severe COVID-19 pneumonia by the addition of nafamostat to standard-of-care [16]. A retrospective observational study also failed to find an association between nafamostat mesylate and in-hospital mortality in patients with COVID-19 [17]. 

**Figure 1:**
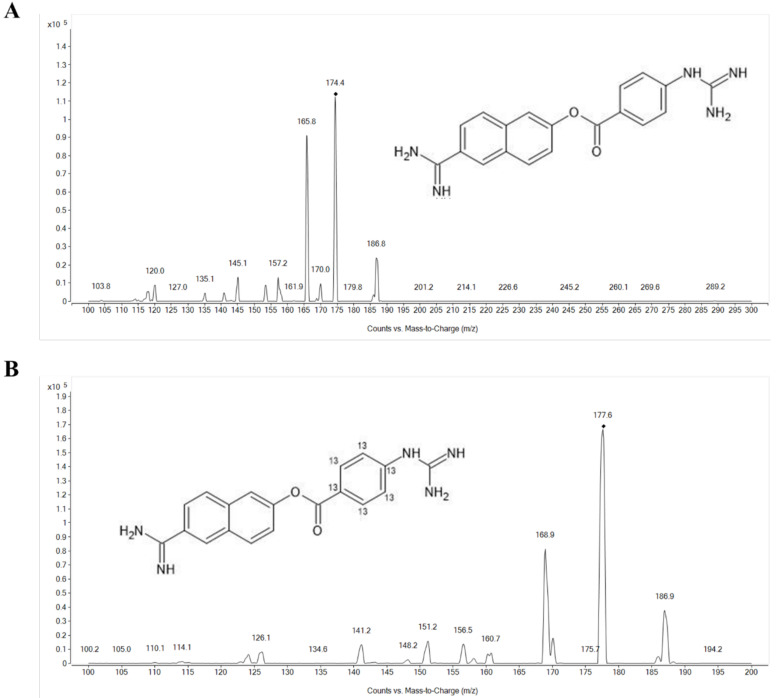
Product ion spectra of (**A**) protonated nafamostat ([M + 2H]^2+^, *m*/*z* = 174.4) and (**B**) ^13^C_6_-nafamostat (IS) ([M + 2H]^2+^, *m*/*z* = 177.4).

Despite its long-term pharmacological use and potential utility for COVID-19, however, there are only a few studies that describe the analysis of nafamostat in the biological fluids for in vivo pharmacokinetic studies [18]. The challenges in the bioanalysis of nafamostat are mainly associated with its highly polar and unstable properties. Nafamostat (6′-amidino-2-naphthyl 4-guanidinobenzoate) is an ester conjugate of 6-amidino-2-naphthol and p-guanidinobenzoic acid (Figure 1A). The ester structure of nafamostat has been recognized as an essential moiety for its pharmacological activity [19]. Nevertheless, the ester conjugate is highly unstable because it is rapidly hydrolyzed into 6-amidino-2-naphthol and p-guanidinobenzoic acid by the esterase that is present throughout the body [20]. Moreover, nafamostat possesses prominent polar groups such as guanidine that contribute to its high polarity [21]. It is well recognized that the bioanalysis of polar compounds is challenging due to poor retention on reversed-phase columns, significant matrix effect, potential endogenous interference, and poor recovery and sensitivity [21,22]. Although radioisotope-labeling [23], spectrofluorometry based on trypsin-inhibitory activity [4], and liquid chromatography (LC)–UV [20] methods have been reported, they are prone to overestimate the results, are time-consuming, or have poor sensitivity, which hampers their practical application to in vivo pharmacokinetic studies of nafamostat. There is only an LC-MS method that has been developed to quantitate nafamostat in human plasma and evaluate its pharmacokinetics in human volunteers [18]. Thus, detailed preclinical and clinical pharmacokinetic information on nafamostat is still very limited and a sensitive and robust bioanalytical method is indispensable. The analytical method and a better understanding of the pharmacokinetics would be fundamental for the further evaluation of nafamostat as a new treatment option for COVID-19. 

Therefore, this study aimed to develop and validate a simple and sensitive liquid chromatography–tandem mass spectrometry (LC-MS/MS) method for nafamostat and evaluate the pharmacokinetics in rats. The intrinsic selectivity of LC-MS/MS with the optimized sample preparation method allowed us to accurately determine the concentration of nafamostat in the rat plasma with superior sensitivity compared to previous methods. The developed LC-MS/MS method has been successfully applied to assess the pharmacokinetics and oral bioavailability of nafamostat in rats.

## 2. Results and Discussion

An LC-MS/MS assay for the determination of nafamostat in the rat plasma was developed and validated. Since nafamostat is polar and highly unstable in plasma, the plasma sample preparation and solid phase extraction (SPE) method were optimized to improve the extraction efficiency and sensitivity. Application of the developed LC-MS/MS method was demonstrated through in vivo pharmacokinetic studies of nafamostat in rats.

Conventional methods including radioisotope-labeling [23], spectrofluorometry based on trypsin-inhibitory activity [4], and LC-UV [20] methods for nafamostat have been reported. However, they have limitations when applied to in vivo pharmacokinetic studies. For example, the radioisotope-labeling method [23] cannot distinguish the parent and the metabolites if the metabolites also contain radioisotope, leading to the overestimation of the parent drug. The utility of spectrofluorometry [4] and LC-UV [20] methods are also limited due to the time-consuming preparation and low sensitivity when applied to in vivo pharmacokinetic studies. Mass spectrometry-based analyses are also available, but they have been developed for the analysis of nafamostat along with other highly polar compounds in the environmental water [24] or for identification of impurities [25], not for in vivo studies. There are only a few LC-MS methods available for in vivo pharmacokinetic studies [18]. The present LC-MS/MS method further optimized sample extraction and preparation methods for nafamostat, resulting in a robust analysis of nafamostat in the plasma with superior sensitivity (LLOQ = 0.5 ng/mL).

### 2.1. Mass Spectrometry

**Figure 2:**
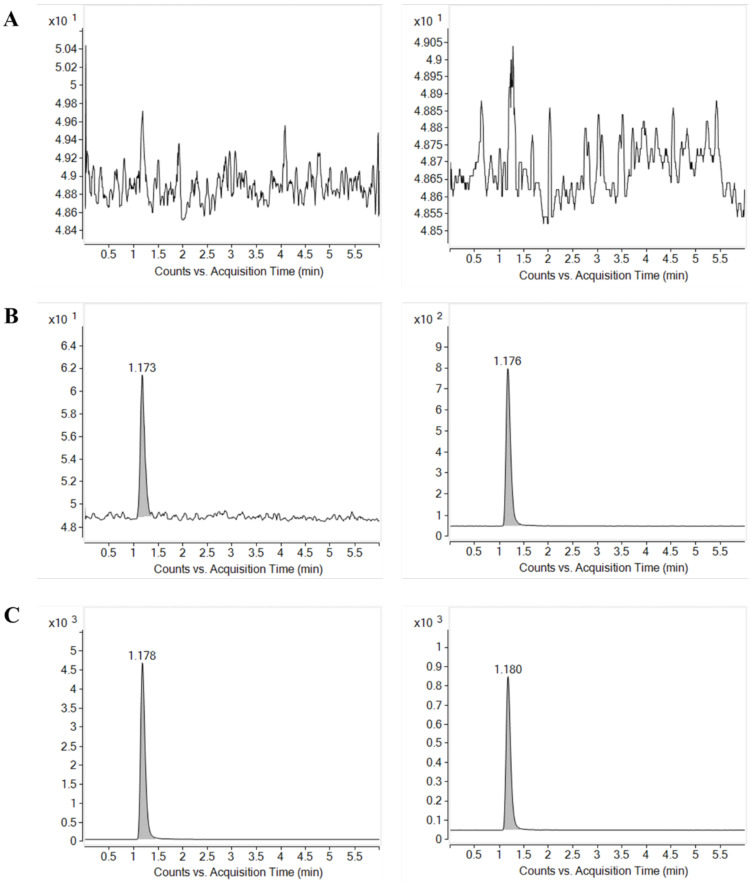
Multiple reaction monitoring (MRM) chromatograms of nafamostat (left) and IS (right) obtained from (**A**) blank rat plasma, (**B**) blank rat plasma spiked with LLOQ concentration of nafamostat (0.5 ng/mL) and IS, and (**C**) blank rat plasma spiked with ULOQ concentration of nafamostat (200 ng/mL) and IS.

The product ion mass spectra of protonated nafamostat and the internal standard (IS), ^13^C_6_-nafamostat, are shown in Figure 1. As the molecular weight of nafamostat is 347.37, the most abundant ion was [M + 2H]^2+^ at *m*/*z* 174.4 for nafamostat and [M + 2H]^2+^ at *m*/*z* 177.4 for IS in the positive Q1 mass scan spectrum. The most prominent fragment ion of the protonated nafamostat and IS were *m/z* 165.8 and *m*/*z* 168.9, respectively (Figure 1). Consequently, the multiple reaction monitoring (MRM) transitions of *m*/*z* 174.4 → 165.8 for nafamostat and *m*/*z* 177.4 → 168.9 for IS were selected and monitored. Representative MRM chromatograms of nafamostat and IS obtained by extraction of blank plasma spiked with nafamostat and IS are shown in Figure 2. The retention times of nafamostat and IS in the plasma were both 1.17 min.

### 2.2. Solid Phase Extraction

Due to its high polarity and instability, effective extraction of nafamostat from biological fluids with minimized hydrolysis is critical for a successful analysis [18]. It has been reported that protein precipitation and liquid–liquid extraction with various solvents did not result in good extraction recovery or sensitivity [18,26]. We also tried protein precipitation with acetonitrile or methanol to extract nafamostat from plasma samples, but a significant matrix effect was observed. 

Therefore, the SPE method was developed and optimized. To optimize the SPE method, various washing and eluting solvents including formic acid and ammonium hydroxide were examined. Table 1 shows the peak response of nafamostat in the rat plasma (100 ng/mL) extracted by SPE method using various washing solvents and eluting solvents. While the impact of washing solvent was less significant, the pH of the eluting solvent in SPE significantly affected the peak response of nafamostat. Plasma samples washed with 0.1% aqueous formic acid and eluted with methanol in SPE showed the greatest peak area and the lowest SD and was selected as the optimal SPE method for plasma sample preparation. Finally, the plasma samples prepared by the optimized SPE method show the average extraction recovery of over 82.58% for nafamostat and 75.28% for ^13^C_6_-nafamostat. Although the recovery percentages slightly deviated from each other, the extent of the recovery of both nafamostat and ^13^C_6_-nafamosat was consistent as recommended by FDA guidance [27]. 

### 2.3. Plasma Sample Preparation and Storage

Nafamostat in the biological fluid is highly unstable because it is rapidly hydrolyzed by erythrocytes in the blood, as well as by arylesterase in the plasma [20]. Quick plasma preparation is one of the general stabilization strategies for unstable compounds in the biological matrices [18,28]. Therefore, to prevent the hydrolysis of nafamostat by erythrocytes after acquiring the blood samples, the centrifugation time of the blood samples was shortened from 10 to 5 min. In addition, the stability of nafamostat was examined by modulating the pH of plasma samples to determine the optimal pH to minimize the enzymatic hydrolysis.

Table 2 shows the stability of nafamostat in the plasma at different pH, (1) 0.35% HCl (pH 1.2); (2) 0.035% HCl (pH 1.9); (2) 1.0% formic acid (pH 2.2); (3) 0.1% formic acid (pH 2.7); (4) saline (pH 5.5); (5) 0.1% ammonium hydroxide (pH 10.5) by adding 2.0 vol% of the reagents. Nafamostat concentrations were also determined under various storage conditions, i.e., (A) immediately, (B) after 24 h left at room temperature, (C) after being subjected to five freeze–thaw cycles, and (D) after 10 days at −20 °C. As shown in Table 2, the low pH conditions, i.e., 0.35% HCl and 1.0% formic acid, maintained nafamostat stability in the plasma under all tested conditions. On the other hand, the stability of nafamostat at higher pH was significantly reduced, especially at room temperature for 24 h. These data indicate that an acidic pH was essential to inhibit enzymatic hydrolysis of nafamostat in the plasma samples. Accordingly, once plasma samples were collected, 3 µL of 5.5 mol/L HCl was added to 150 µL of the collected plasma samples to keep the plasma samples in 0.35% HCl (pH = 1.2).

As indicated in the optimization of SPE process (Table 1) as well, these data suggested that pH control is critical for the analysis of nafamostat. Since most enzymatic or non-enzymatic reactions occur in a favorable pH range, a proper pH control during the entire analytical process is required to minimize the degradation of unstable compounds [28].

### 2.4. Method Validation

#### 2.4.1. Specificity, Linearity, and Sensitivity

As shown in Figure 2, examination of blank plasma and blank plasma spiked with IS and nafamostat at the lower limit of quantification (LLOQ = 0.5 ng/mL) and upper limit of quantification (ULOQ = 200 ng/mL) indicated no interfering endogenous or exogenous peaks at the retention time corresponding to nafamostat and IS. The absence of interfering components in the chromatograms for nafamostat demonstrated the specificity of the present method. 

The calibration curve of nafamostat was linear over the calibration standard concentration range from 0.5 to 200 ng/mL with a correlation coefficient >0.999. The LLOQ was 0.5 ng/mL, which was defined as the lowest standard concentration on the calibration curve. The signal-to-noise (S/N) ratio of the nafamostat peak at LLOQ was 104.7.

#### 2.4.2. Extraction Recovery

Table 3 shows the extraction recovery of nafamostat at low, medium, and high matrix-matched quality control (QC) samples and IS. The extraction recovery was calculated as the ratio of the peak area obtained from standard solution spiked in pre-extraction to those in post-extraction. The average recoveries of extraction for nafamostat in the rat plasma at low QC (2 ng/mL), medium QC (80 ng/mL), and high QC (160 ng/mL) levels were 83.44%, 82.58%, and 89.28%, respectively. The average extraction recovery for IS was 75.28%. These data indicate that the extraction of nafamostat in the rat plasma was efficient and reproducible.

#### 2.4.3. Accuracy and Precision

Table 4 shows the intra- and inter-day accuracy and precision determined at the LLOQ (0.5 ng/mL), low, medium, and high QC concentrations with five replicates each day for five consecutive days. The intra- and inter-day accuracies were 95.78–105.16%. The precisions were all within 7.91%. The intra- and inter-day accuracy and precision of the present assay satisfied the criteria of the FDA guidance on bioanalytical methods validation [27].

#### 2.4.4. Stability

The stability of the nafamostat in the rat plasma was determined under four different storage conditions. Table 5 shows the autosampler stability, freeze–thaw stability, short-term and long-term stability of nafamostat in the rat plasma. No significant deviations in the measured concentrations under any tested conditions were observed compared to the freshly prepared samples, indicating that the nafamostat was stable for application in the routine analysis.

#### 2.4.5. Dilution Integrity

Dilution integrity was examined by evaluating the accuracy and precision of the plasma sample that was spiked with 2000 ng/mL of nafamostat and diluted 10 times with blank plasma (*n* = 5). The average accuracy and precision were 106.69% and 3.28%, respectively, which were both within the set criteria (±15%), indicating that dilution of plasma samples did not affect the accuracy and precision. 

### 2.5. Pharmacokinetics of Nafamostat in Rats

The developed LC-MS/MS assay was applied to pharmacokinetic studies to characterize the pharmacokinetics and bioavailability of nafamostat following intravenous and oral administration in rats. All protocols in the animal study were approved by the Ethics Committee for the Treatment of Laboratory Animals at Sungkyunkwan University (SKKUIACUC2021-01-57-1) and conducted in accordance with the standard operating procedures (SOPs). 

Despite the versatile pharmacological activities of nafamostat and its potential as a treatment option for COVID-19, the pharmacokinetic information of nafamostat is very limited. Although a clinical pharmacokinetic study after intravenous infusion is available [18], comprehensive pharmacokinetic characterization of nafamostat, including the oral bioavailability, has not been reported.

**Figure 3:**
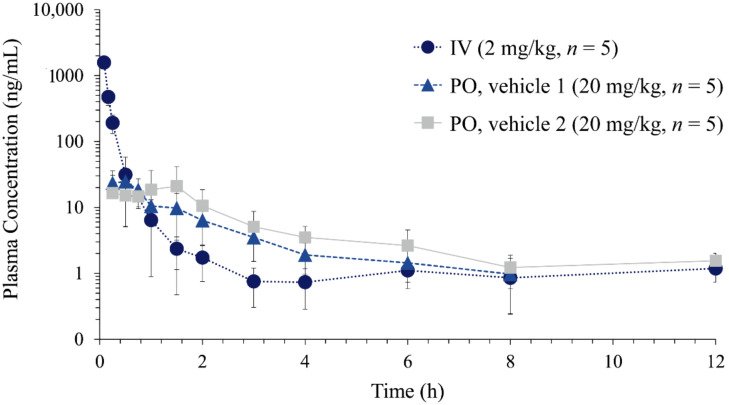
Plasma concentration vs. time profiles of nafamostat following intravenous injection (IV) of nafamostat dissolved in 5% DMSO (2 mg/kg, *n* = 5) and oral administration (PO) dissolved in vehicle 1 (10% DMSO, 20 mg/kg, *n* = 5) and vehicle 2 (10% DMSO and 10% Tween 80, 20 mg/kg, *n* = 5) in rats.

Therefore, nafamostat dissolved in 5% DMSO was administered by intravenous injection via penile vein at a dose of 2 mg/kg (*n* = 5). For oral administration, nafamostat was dissolved in two types of vehicles: (1) 10% DMSO, (2) 10% DMSO with 10% Tween 80, and orally administered at a dose of 20 mg/kg (*n* = 5). Figure 3 shows the average plasma concentration–time profiles of nafamostat after intravenous injection (2 mg/kg) and oral administration (20 mg/kg). The non-compartmental pharmacokinetic parameters of nafamostat are summarized in Table 6. After intravenous injection of nafamostat at a dose of 2 mg/kg, the plasma nafamostat concentration–time profile declined in a multi-exponential manner with an average terminal half-life (t_1/2_) of 1.34 ± 0.51 h. The area under the curve (AUC_inf_), systemic clearance (CL), and steady-state volume of distribution (V_ss_) was 447.35 ± 35.93 ng∙h/mL, 74.91 ± 6.11 mL/min/kg, and 0.99 ± 0.65 L/kg, respectively. The t_1/2_ of nafamostat observed in rats was comparable to the reported t_1/2(β)_ in humans, i.e., 112–128 min [18]. 

Following oral administration, nafamostat was rapidly observed and reached its peak concentration (C_max_) within approximately 1.0 h. The average oral bioavailability of nafamostat was 0.95% and increased to 1.59% when Tween 80 was included in the dosing vehicle (Table 6). Although the number of hydrogen bond donors is 7, nafamostat has the molecular weight (347.37) < 500, LogP (2.062) < 5, and hydrogen bond acceptors (7) < 10, satisfying Lipinski’s rule [29]. The topological polar surface area of 138 Å^2^ and rotatable bond count of 5 also does not violate Veber’s rule for toxicity [30]. These predictions agree with the good safety profile of nafamostat and rapid absorption after oral administration.

The rate of absorption depends on how rapidly the drug is absorbed across the gut wall into the systemic circulation. On the other hand, the extent of absorption depends on the overall fraction of gut wall permeability and first-pass metabolism in the gastrointestinal tract and liver. Although a drug rapidly penetrates the gut wall, the drug may be extensively metabolized either in the gut wall or in the liver before it reaches the systemic circulation, leading to low bioavailability. Moreover, nafamostat is extensively hydrolyzed by esterases that are present throughout the body, which would also contribute to the low bioavailability. The low oral bioavailability may be one of the reasons why the oral formulation of nafamostat is not available. Nevertheless, oral nafamostat has shown pharmacological activity [31,32], and the development of structurally related compounds with improved oral bioavailability has been continuously pursued [33]. A recently completed Phase 1 clinical trial (NCT04406415) also evaluated the safety, tolerability, and pharmacokinetics of oral nafamostat in healthy volunteers.

Tween 80 (Polysorbate 80) is a hydrophilic nonionic surfactant that is widely used for various drug products as an emulsifier, stabilizer, wetting agent, foaming agent, or dispersant [34]. By enhancing the permeability of phospholipid membranes, Tween 80 improves skin penetration for transdermal drug delivery [35,36]. Tween 80 is also known to improve the bioavailability of a drug after oral administration [37,38,39,40]. Tween 80 may modulate the intestinal membrane permeability leading to improved oral bioavailability [40]. Studies have indicated that Tween 80 may inhibit efflux transporters such as P-glycoprotein (P-gp), multidrug resistance-associated protein (MRP2), and breast cancer resistance protein (BCRP) and improve oral absorption [38,39,41]. The low oral bioavailability of nafamostat is likely due to its high polarity, which would limit gastrointestinal permeability. Although the effect was not substantial, the addition of a surfactant, i.e., Tween 80, increased the systemic exposure represented by C_max_ and AUC_inf_, as well as the bioavailability potentially by modulating the intestinal membrane permeability. There was also a trend of extended time to reach C_max_, i.e., T_max_, after oral administration. Further studies are required to demonstrate the potential improvement of the oral bioavailability of nafamostat via formulation development.

## 3. Materials and Methods

### 3.1. Materials

Nafamostat mesylate was purchased from Tokyo Chemical Industry Co. (Tokyo, Japan). Stable isotope-labeled internal standard (IS), ^13^C_6_-Nafamostat, was purchased from Alsachim Co. (Illkirch Graffenstaden, France). Hydrochloric acid was purchased from Merck KGaA (Darmstadt, Germany). Formic acid and ammonium hydroxide were the products of Sigma-Aldrich Co. (St. Louis, MO, USA). High-performance liquid chromatography (HPLC)-grade methanol and distilled water were purchased from J.T. Baker, Inc. (Phillipsburg, NJ, USA).

### 3.2. Sample Preparation

#### 3.2.1. Stock Solutions, Calibration Standards, and Quality Control Samples

Stock solutions of nafamostat and IS (^13^C_6_-nafamostat) were prepared by dissolving 7.8 mg of nafamostat mesylate in 10 mL of methanol and 1.054 mg of ^13^C_6_-nafamostat formate in 1.67 mL of methanol, respectively. The stock solution was serially diluted with methanol, yielding concentrations of 0.5, 1, 5, 10, 20, 50, 100, and 200 ng/mL of standard working solutions. The IS working solution (100 ng/mL) was prepared by diluting the stock solution with methanol.

Calibration samples were prepared by spiking 50 μL of the standard working solution and 50 μL of the IS working solution to 50 μL of the rat blank plasma and 850 μL of 0.1% (*v/v*) aqueous formic acid. The mixture was mixed on a vortex mixer for 1 min. Quality control (QC) samples were prepared by spiking the working standard solutions to the rat blank plasma to provide high concentration QC (160 ng/mL), medium concentration QC (80 ng/mL), low concentration QC (2 ng/mL), and lower limit of quantification (LLOQ) QC (0.5 ng/mL). The QC samples were stored at −70 °C until analysis.

Plasma samples were also prepared by adding the IS working solution 50 μL and methanol 50 μL to 50 μL of the obtained plasma samples followed by the addition of 850 μL of 0.1% (*v/v*) aqueous formic acid. The mixture was then mixed in a vortex mixer for 1 min.

#### 3.2.2. Sample Extraction

The samples were extracted by solid phase extraction (SPE) on Oasis HLB 1cc (10 mg) extraction cartridge (Waters, Milford, MA, USA). The extraction cartridge was conditioned with 2 mL of methanol followed by 1 mL of distilled water. The sample (900 µL) was then loaded into the cartridge and washed with 1 mL of 0.1% (*v/v*) aqueous formic acid. Finally, 1 mL of methanol was used as an eluting solvent, and the elute was reconstituted in 1 mL of distilled water. An aliquot (3 μL) was injected onto the LC-MS/MS.

### 3.3. LC-MS/MS Conditions

The LC-MS/MS analysis was performed by Agilent 6490 (Agilent Technologies, Santa Clara, CA, USA) coupled with Agilent 1260 HPLC system (Agilent Technologies). The analyte was separated on a Gemini 5 μm C18 110A column (150 × 2 mm i.d., 5 μm, Phenomenex, Torrence, CA, USA) with SecurityGuard Cartridge Kit (Phenomenex). An isocratic mobile phase composed of 0.1% aqueous formic acid and methanol (50:50 *v/v* %) was used with a flow rate of 0.3 mL/min. The column oven temperature was 40 °C and the total run time was 6 min.

The electrospray ionization (ESI) source was operated in positive mode, and the mass spectrometer was operated in the multiple reaction monitoring (MRM) mode with a dwell time of 200 ms per MRM channel. Gas temperature, gas flow rate, and nebulizer gas pressure were set at 220 °C, 17 L/min, and 45 psi, respectively. The selected precursor/product ion pairs were *m*/*z* 174.4 → 165.8 for nafamostat and 177.4 → 168.9 for IS. The fragment voltage was 380 V, and the collision energy was set at 11 eV for both nafamostat and IS. The mass spectrometric data were processed by MassHunter Quantitative Analysis (Agilent Technologies).

### 3.4. Assay Validation

#### 3.4.1. Specificity, Linearity, and Sensitivity

Specificity was assessed by analyzing the blank matrix, and the blank matrix spiked with the nafamostat and IS. The linearity of the method was evaluated over the concentration ranges of 0.5–200 ng/mL in the rat blank plasma. The calibration curves were constructed by the weighted regression method (1/×) of peak area ratios of the analyte to IS vs. actual concentration. The lower limit of quantification (LLOQ) was defined as the lowest analyte concentration of the calibration range, which yielded the analyte response five times higher than that of the zero calibrators with acceptable accuracy and precision ≤20%.

#### 3.4.2. Accuracy and Precision

Accuracy and precision were determined by assaying five replicates of each LLOQ, and low, medium, and high QC samples on the same day (intra-day) and five consecutive days (inter-day). Accuracy was calculated as the percentage of the mean back-calculated concentration versus nominal concentration. Precision was calculated as the coefficient of variance of each concentration. Acceptable criteria for accuracy and precision were within ±15% relative error from the nominal values and within ±15% relative standard deviation except at LLOQ, where it should not deviate by more than 20% [27].

#### 3.4.3. Extraction Recovery

Extraction recoveries of the nafamostat and IS were determined by using three replicates of low, medium, and high QC samples. The extraction recovery was calculated by comparing the peak responses of the analyte in samples spiked before and after the extraction.

#### 3.4.4. Stability

The stability of nafamostat and IS was examined under four different conditions using three replicates of low, medium, and high QC samples in the rat plasma. The autosampler stability was determined by analyzing the QC samples left in the autosampler at 4 °C for 24 h. The freeze–thaw stability was determined by analyzing the QC samples subject to five freeze–thaw cycles. The short-term stability was determined by analyzing the QC samples left at room temperature for 24 h. Finally, the long-term stability was determined by analyzing the low, medium, and high QC samples left at −70 °C for 2 weeks. All stability data were expressed as the percentage of the mean response of the QC samples for stability tests vs. those of the freshly prepared samples at the same concentrations.

#### 3.4.5. Dilution Integrity

Dilution integrity was examined at 2000 ng/mL, which was ten times higher than the upper limit of plasma calibrators. The samples were diluted with blank plasma, and the concentration was measured (*n* = 5). The actual concentration was then calculated by multiplying the dilution factor (×10) to the measured concentration. The precision was expressed as the coefficient of variation, and the accuracy was expressed as the percentage of the mean calculated vs. actual concentrations.

### 3.5. Pharmacokinetic Studies of Nafamostat in Rats

The animal study was approved by the Ethics Committee for the Treatment of Laboratory Animals at Sungkyunkwan University (SKKUIACUC2021-01-57-1) and conducted in accordance with the standard operating procedures (SOPs). Male Sprague–Dawley rats (7 weeks, 188–204 g; DBL, Eumseong, Korea) were kept in plastic cages with free access to a standard diet (Cargill Agri Purina, Seongnam, Korea) and water. The animals were maintained at a temperature of 23 ± 2 °C with a 12 h light–dark cycle and relative humidity of 50 ± 10%.

Nafamostat dissolved in 5% DMSO (1 mg/mL) was administered by intravenous injection via penile vein at a dose of 2 mg/kg (*n* = 5). Approximately 0.3 mL of the venous blood samples were collected at 5, 10, 15, 30 min, 1, 1.5, 2, 3, 4, 6, 8, and 12 h after drug administration directly from the jugular vein. For oral administration, nafamostat was dissolved in two types of vehicle: (1) 10% DMSO, (2) 10% DMSO with 10% Tween 80. The prepared nafamostat solution in Vehicle 1 or Vehicle 2 was orally administered at a dose of 20 mg/kg (*n* = 5). Approximately 0.3 mL of the venous blood samples were collected at 15, 30, 45 min, 1, 1.5, 2, 3, 4, 6, 8, and 12 h after drug administration directly from the jugular vein. Plasma samples were obtained by centrifugation of the blood samples at 15,000× *g* for 5 min. Finally, 150 μL of the plasma samples were mixed with 3 μL of the 5.5 mol/L HCl and stored at −20 °C until analysis.

The plasma nafamostat concentration–time data were analyzed by a non-compartmental method using the nonlinear least-squares regression program, Phoenix WinNonlin (Version 6.4, Certara, L.P., Princeton, NJ, USA).

## 4. Conclusions

A rapid, simple, and sensitive bioanalysis of nafamostat by using LC-MS/MS was developed and validated. Optimization of the sample preparation and SPE method enabled enhanced extraction recovery and improved assay sensitivity. Since the present method does not involve any time-consuming processes, i.e., derivatization or nitrogen gas drying, the present SPE method is relatively simpler than previous sample preparation methods for nafamostat. Finally, the developed LC-MS/MS assay provided minimal degradation of nafamostat and a sensitivity of 0.5 ng/mL in the rat plasma, which allowed full characterization of the in vivo pharmacokinetics and oral bioavailability of nafamostat in rats. Pharmacokinetic information can help to determine appropriate doses for clinical studies and to make decisions regarding formulation design and dosing regimens, which is critical for successful drug development or repositioning. A sensitive and robust analytical method to accurately quantitate drug concentrations in biological fluid is fundamental for pharmacokinetic studies. In particular, for drugs that are unstable and have low bioavailability, such as nafamostat, a sensitive analytical method is essential to accurately determine the half-life in the terminal phase and obtain pharmacokinetic information. Therefore, the present LC-MS/MS method and pharmacokinetic information would be helpful for further preclinical and clinical studies of nafamostat. 

## Figures and Tables

**Table 1:** The effect of washing solvent and eluting solvent by SPE method on the peak area of nafamostat (100 ng/mL) in the rat plasma (mean ± SD, *n* = 3).

		Washing Solvent			
		1% Formic Acid	0.1% Formic Acid	Distilled Water	0.1% Ammonium Hydroxide
Eluting Solvent	1% formic acid in methanol	31,842 ± 1914	33,517 ± 733	30,554 ± 3255	35,875 ± 411
	0.1% formic acid in methanol	30,784 ± 2930	34,495 ± 227	34,259 ± 4558	37,565 ± 4206
	Methanol	32,590 ± 1869	36,666 ± 397	35,188 ± 2040	-
	0.1% ammonium hydroxide in methanol	25,608 ± 2646	4686 ± 2211	-	-

**Table 2:** Effects of pH on the stability (%) of nafamostat 160 ng/mL in the plasma under different conditions (mean ± SD, *n* = 3).

Storage Condition	0.35% HCl (pH = 1.2)	1.0% Formic Acid (pH = 2.2)	0.1% Formic Acid (pH = 2.8)	Saline (pH = 5.5)	0.1% Ammonium Hydroxide (pH = 10.5)
A	103.67 ± 0.60	97.66 ± 6.58	66.56 ± 3.73	63.47 ± 2.32	29.29 ± 1.26
B	104.42 ± 2.50	100.05 ± 1.83	0.16 ± 0.05	0.13 ± 0.03	0.09 ± 0.01
C	103.12 ± 2.19	101.86 ± 1.51	48.25 ± 2.26	19.01 ± 1.61	5.21 ± 0.32
D	107.15 ± 0.69	105.67 ± 0.59	0.12 ± 0.07	9.93 ± 1.76	0.13 ± 0.08

A, immediately; B, after 24 h left at room temperature; C, after being subjected to 5 freeze-thaw cycles; D, after 10 days at −20 °C. Stability (%) was expressed as the observed concentration compared to the theoretical concentration.

**Table 3:** Extraction recovery of nafamostat and ^13^C_6_-nafamostat (IS) in the rat plasma (mean ± SD, *n* = 3).

Compound	Concentration (ng/mL)	Extraction Recovery (%)
Nafamostat (*n* = 3)	2	83.44 ± 5.22
	80	82.58 ± 2.69
	160	89.28 ± 2.38
^13^C_6_-nafamostat (*n* = 9)	100	75.28 ± 6.33

**Table 4:** Intra- and inter-day accuracy and precision of nafamostat in the rat plasma (mean ± SD, *n* = 5).

Concentration (ng/mL)	Intra-Day (*n* = 5)			Inter-Day (*n* = 5)		
	Concentration Found (ng/mL)	Accuracy (%)	Precision (%)	Concentration Found (ng/mL)	Accuracy (%)	Precision (%)
0.5	0.49 ± 0.04	97.75	7.91	0.52 ± 0.03	104.44	4.92
2	2.10 ± 0.04	105.16	1.69	2.05 ± 0.14	102.68	6.71
80	76.63 ± 1.03	95.78	1.34	81.89 ± 3.29	102.37	4.01
160	163.12 ± 3.95	101.95	2.42	160.47 ± 1.46	100.30	0.91

**Table 5:** Stability of nafamostat in the rat plasma (mean ± SD, *n* = 3).

Concentration (ng/mL)	Percentage over Theoretical Concentration (%)			
	Autosampler Stability (24 h, 4 °C)	Freeze/Thaw Stability (5 Cycles, −20 °C)	Short-Term Stability (24 h, 20 °C)	Long-Term Stability (2 weeks, −20 °C)
2	97.2 ± 4.98	99.02 ± 3.61	96.57 ± 2.66	97.25 ± 4.73
80	101.31 ± 2.00	100.40 ± 0.20	101.11 ± 1.95	99.38 ± 0.49
160	97.45 ± 0.31	98.44 ± 0.58	97.64 ± 2.18	96.62 ± 1.63

**Table 6:** Pharmacokinetic parameters of nafamostat after intravenous (IV) injection (2 mg/kg) and oral administration (20 mg/kg) of nafamostat in rats (mean ± SD, *n* = 5).

Parameters	IV Injection (2 mg/kg, *n* = 5)	Oral Administration (20 mg/kg, *n* = 5)	
		Vehicle 1: 10% DMSO	Vehicle 2: 10% DMSO + 10% Tween 80
t_1/2_ (h)	1.34 ± 0.51	2.21 ± 1.30	2.30 ± 1.11
C_0_ or C_max_ (ng/mL)	5291.16 ± 808.04	27.76 ± 8.17	33.24 ± 18.76
T_max_ (h)	-	0.45 ± 0.21	1.00 ± 0.53
AUC_inf_ (ng·h/mL)	447.35 ± 35.93	42.43 ± 12.50	71.17 ± 29.91
V_z_ or V_z_/F (L/kg)	8.75 ± 3.80	1906.08 ± 1841.14	998.85 ± 401.04
CL or CL/F (mL/min/kg)	74.91 ± 6.11	8558.92 ± 3081. 60	5321.71 ± 1985.58
V_ss_ (L/kg)	0.99 ± 0.65	-	-
Bioavailability		0.95 ± 0.25%	1.59 ± 0.60%

## Data Availability

The data presented in this study are available on request from the corresponding author.
